# Counting the dead and making the dead count: configuring data and accountability

**DOI:** 10.1007/s40656-021-00415-5

**Published:** 2021-04-26

**Authors:** Brian Rappert

**Affiliations:** Department of Sociology, Philosophy and Anthropology, University of Exeter, Exeter, EX4 4RJ UK

**Keywords:** Ignorance, Accountability, Excess deaths, Covid-19, Iraq, Data

## Abstract

This article examines the relation between counting, counts and accountability. It does so by comparing the responses of the British government to deaths associated with Covid-19 in 2020 to its responses to deaths associated with the 2003 invasion of Iraq. Similarities and dissimilarities between the cases regarding what counted as data, what data were taken to count, what data counted for, and how data were counted provide the basis for considering how the bounds of democratic accountability are constituted. Based on these two cases, the article sets out the metaphors of leaks and cascades as ways of characterising the data practices whereby counts, counting and accountability get configured. By situating deaths associated with Covid-19 against previous experience with deaths from war, the article also proposes how claims to truth and ignorance might figure in any future official inquiry into the handling of the pandemic.

## Introduction

Data fracture. Historical, political and sociological studies of science have explored the contrasting but often co-existing characterisations of data: given and made (Leonelli, 2016) as well as speaking for themselves and needing to be spoken for (Latour, 1987). Their evidential standing is frequently not just about the status of data per se, but bound up with the identity of those that hold and notionally generate them (Shapin, 1994). The adage ‘Knowledge is power’ speaks to enduring tenets in popular culture and academic scholarship alike. In line with the doubleness that so often characterises data and knowledge, however, claims to ignorance and uncertainty have likewise been identified as bases for establishing epistemic warrant and exercising political control (Gross & McGoey, 2015; Proctor & Schiebinger, 2008).

As part of attempts to scale the Covid-19 pandemic, notable efforts have been undertaken to determine the number of deaths associated with the virus. While such aggregates provide a comparatively trackable measure of harm, their accuracy and reliability have also been recurring topics of debate.^1^ The multi-faceted status of deaths tallies—actual and protean, given and made—is the focus of this article. Its central aim is to establish how the mobilisation and communication of data on deaths constitute and delimit democratic accountability.

The chief method for doing so is comparison. As explored by Musu (2020), as with past diseases and other crises, the relationship between human populations and Covid-19 has often been likened to an armed conflict. War has served as an organising analogy for imagining who and what is involved, what is at stake as well as what needs to be done. Rather than approaching Covid-19 through the refractions of this analogy, I offer an alternative basis for analysis that still draws on war, namely: juxtaposing how knowledge and ignorance are alternatively marshalled in pandemics and armed conflicts. Specifically I compare the responses of British government officials^2^ to deaths associated with Covid-19 during the first wave of the pandemic in 2020 to the responses of officials to deaths from the invasion of Iraq in 2003.

To propose comparison as the method for analysis only signals what direction will be taken in the most preliminary sense. Comparison is often identified as ‘troubled’ methodology (Brettell, 2009) because it entails reducing the complexity of cases in favour of being able to pull out certain similarities and differences (Deville et al., 2016). As a result, comparative studies are invariably accompanied by questions: What should be compared to what? On which basis? For what purpose?

This article compares Covid-19 and the Iraq war in relation to how officials accounted for deaths. In particular, it examines the relationship between tallied deaths counts, the counting of deaths and attempts to stymie political accountability for deaths. It elaborates two metaphors for characterising the relation between counts, counting and accountability: *cascades* and *leaks*. By cascades, I refer to data practices in which data are successively refined, but the very dynamism of that refinement is positioned as a basis for treating data as provisional. This metaphor characterises the manner in which accountability for tallied deaths has been forestalled despite the considerable efforts made to count deaths associated with Covid-19. By leaks I refer to data practices in which partialness in the assemblage of data is positioned as the basis for arguing against the prospects for assembling data. This metaphor characterises the manner in which British officials both refuted specific death counts as well as the ability to count deaths in the case of the Iraq war.

In making clear the indeterminate relation between data, counting, counts and accountability, this argument illustrates how claims to knowledge and ignorance can interweave in statecraft. The notions of cascades and leaks are offered as complements to other prominent metaphors today that likewise speak to the link between data and accountability; such as the notions of ‘data dumps’ and ‘data avalanches’.

As will be further contended, the metaphors of leaks and cascades are not only helpful in characterising state actions and inactions, they signal the demands faced by scholars and others in conducting second-order analyses of data practices.

### Structure of argument

As a response to potential concerns about the how comparative cases are likened and differentiated, this article moves between drawing out similarities and differences. The next section begins by attending to similarities between responses to Covid-19 and the Iraq war. In particular, it identifies how officials argued for the inability to establish definitive death *counts* in response to calls to answer for what had taken place. Through their appeals to what is unknown, not-yet-known, not-possible-to-know, not-possible-to-know-with-certainty and not-possible-to-assess and so on, in both cases officials crafted what McGoey (2012) termed ‘strategic unknowns’. Section Three then investigates the differences in skills, institutions and infrastructures marshalled to *count* deaths across the cases. Section Four advances the aforementioned metaphors of cascades and leaks to characterise the conditions of possibility for how data is made to matter (or not).

In mapping out possibilities for accountability, a principal purpose of this argument is to support thinking anew. This will take place in two directions: the extensive counting efforts in the case of Covid-19 are used to suggest the highly delimited bounds of political accountability in relation to the past actions taken in relation to the Iraq war. Furthermore, the official discounting of counts in the Iraq war is used to forewarn how the British state could respond to accountability demands for deaths associated with Covid-19. Along these lines, at the time of the drafting of this article, there is much anticipation of a future public inquiry into the government’s handling of the pandemic. The final section proposes some likely fault lines for this kind of truth-seeking and ignorance-producing endeavour.

## Making the dead count

Accountable

1. Required or expected to justify actions or decisions; responsible.
  *‘ministers are accountable to Parliament’*
2. Able to be explained or understood.^3^

### Pandemic deaths in the UK

During the spring of 2020, British ministers and advisors repeatedly offered justifications for imposing and lifting Covid-19 lockdown restrictions. Traditional political forums to hold government to account for its decisions, such as Parliamentary debate and media interviews, were complemented with novel ones such as the daily press briefings at Number Ten Downing Street.^4^ In these settings, ‘questions and answers’ served as a key interactional mode for displaying relations of accountability. Not only were officials interrogated on government policies and practices; they were also able to message to the public its responsibilities during the pandemic (to stay at home, to stay alert, and so on).

**Fig. 1:**
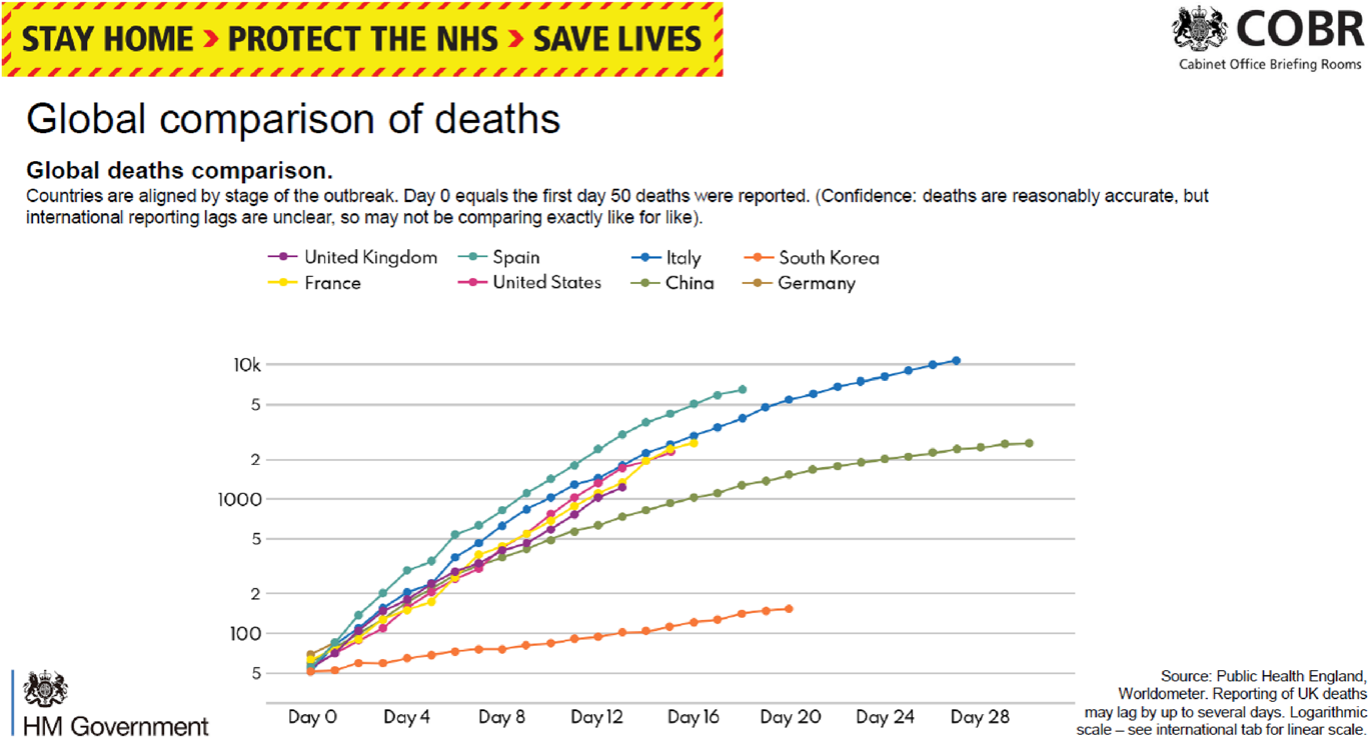
Global Comparison of Deaths on 30 March 2020 Prime Minister’s Office (2020) (Reproduced under Open Government Licence.)

Within these interactions, data—on confirmed cases, hospital admissions, critical care bed patients, and on deaths—functioned as one of the central bases for constituting mutual relations of accountability. Along these lines, counting the dead with a view to assessing government performance was an explicit ongoing topic from the start of the pandemic. For instance, when the Number 10 press briefings began in March 2020, one of the forms of data presented included a slide scaling deaths totals in different countries (see Fig. 1). Excel sheets with the underlying data were posted on the Number 10 website. Accompanying visual depictions of the data provided not only retrospective representations of what had taken place, but the basis for prospective estimates of what might come. Initially such information was interpreted by government officials as pointing to how the spread of the virus in the UK was tracking alongside other countries in Europe (Vallance, 2020).

And yet, more than one connotation of “accountable” is relevant for characterising how data figured into such proceedings. As in the definition at the start of this section, besides referring to the *expectation* to justify actions, the term also refers to the underlying *ability* to explain. By the late spring of 2020, this second connotation was repeatedly made relevant. For instance, on 6 May during Prime Minister’s (PM) Question Time in Parliament, the leader of the opposition Labour Party, Keith Starmer, began by asking:

When the Prime Minister returned to work a week ago Monday, he said that many people were looking at the “apparent success” of the Government’s approach, but yesterday we learned that, tragically, at least 29,427 people in the UK have now lost their lives to this dreadful virus. That is now the highest number in Europe and the second highest in the world. That is not success, or apparent success, so can the Prime Minister tell us: how on earth did it come to this? (Hansard, 2020a: Column 548)

In doing so, Starmer evoked the number of direct deaths officially attributed to the virus to rank the performance of the UK and thereby evaluate the British government. This was done as part of calling into doubt the PM’s previously aired assessment. Prime Minister Johnson replied to Starmer’s call for an explanation by contending that:

First, of course every death is a tragedy and the right hon. and learned Gentleman is right to draw attention to the appalling statistics, not just in this country but around the world. In answer to his question, I would echo what we have heard from Professor David Spiegelhalter and others: at this stage I do not think that the international comparisons and the data are yet there to draw the conclusions that we want […]

Here, rather than offering a justification for his previous assessment of success or explaining what caused the 29,427 deaths, Johnson referred to named and unnamed others to support the contention that conclusions could not yet be drawn based on then present data. As such, no justifications would be given in relation to the ‘appalling statistics’ cited by Starmer because comparisons could not yet be made.

Starmer responded to Johnson’s suggestion by stating:

The argument that international comparisons cannot be made, when the Government have for weeks been using slides such as the one I am holding to make international comparisons, really does not hold water. I am afraid that many people are concluding that the answer to my question is that the UK was slow into lockdown, slow on testing, slow on tracing and slow on the supply of protective equipment. (Hansard, 2020a: Column 548)

Here, the prior availability of information on deaths served as a situational resource for contesting Boris Johnson’s suggestion that the ‘international comparisons and the data are yet there to draw the conclusions that we want’.

This exchange in Parliament was just one of a series of situations in the first half of 2020 (and then well beyond into 2021) in which the government was asked to account for the rising and comparatively high number of deaths in the UK, in which officials responded through what could be characterised as a tactic of *delay* (e.g., Hansard, 2020b: columns 311–312). For instance, take the 30 April Number Ten Downing Street (2020) daily press briefing. Under questioning on comparative deaths from a journalist, the Prime Minister cautioned that it was necessary to ‘wait and see until the end of the epidemic’ on how countries fared. Citing the article by Professor David Spiegelhalter (mentioned by Johnson above), England’s Chief Medical Officer, Professor Chris Whitty, echoed the importance to ‘not go charging into who has won and who has lost’. Caution was warranted since the variations in place for how nations were calculating deaths in and outside of hospitals meant that comparing their performance was ‘largely a fruitless exercise’. Instead, as Boris Johnson contended at the same press briefing, ‘The only real comparison is going to be possible at the end of the epidemic, when you look at total excess deaths.’

The category of ‘excess deaths’ gained popular salience in the UK during the pandemic. Long familiar to public health specialists, establishing the extent of excess deaths involves comparing the total number of deaths over a given time period to what would be expected in the absence of a major stress, such as an infectious disease. The promises of this measure are that it avoids the vagaries of how deaths are attributed to diseases and it captures indirect deaths (e.g., those stemming from the lack of access to health care).

### Armed conflict in Iraq

The import placed on Covid-19 excess deaths will serve as the entry topic for comparing the pandemic to armed conflict. Years previously, the advisability of measuring excess deaths as a gauge of harm figured prominently in British politics in relation to another major national undertaking: the 2003 invasion of Iraq and the post-invasion conflict that followed.

As background, in March 2003 a coalition of military forces from the United States, the UK, Australia and Poland invaded Iraq. In the UK, the invasion was largely justified in terms of the (falsely) alleged Iraqi possession of so-called weapons of mass destruction. The initial decision by British Prime Minister Tony Blair to support the US-led war effort generated significant domestic political opposition prior to the invasion. While the Iraqi military forces were overrun within a matters of weeks and a Coalition Provisional Authority initially put in place to govern, conflict persisted. The military occupation of the country would become opposed by an armed insurgency. In the years after 2003, the ensuing guerrilla war resulted in considerable deaths to both Iraqis as well as the occupying military forces. The mounting deaths generated further opposition to the original invasion in the US, UK and elsewhere. The analysis of the British government response to attempt to establish Iraqi deaths presented in this article is largely drawn from previous research of the author based on documents obtained under the UK Freedom of Information (FoI) Act (Rappert, 2012a, 2012b) as well as the findings of the official public inquiry into the invasion (Iraq Inquiry, 2016).^5^

As part of the overall contests associated with the invasion, attention to civilian death counts in Iraq underwent a series of shifts within the British government. Between 2003 and 2004, internal government deliberations about the possibility of deriving figures on the number of civilians directly killed in the initial combat phase were initiated in response to growing Parliamentary and public concern post invasion (Iraq Inquiry, 2016: 179–194). Such attention, though, did not lead to meaningful government action to determine the number of civilians killed by UK forces, let alone by the conflict as a whole. For instance, while British forces had kept records of Iraqis that had died during its significant military operations, this information was not collated. Instead, governmental officials repeatedly stated in 2003 and early 2004 that there was no reliable way to determine the number of civilians killed (Iraq Inquiry, 2016: 186).

Attention to deaths in Iraq, and in particular to the category of excess deaths, was brought to the fore of political debate in the UK in response to a 2004 excess deaths study by a group at Johns Hopkins University published in *The Lancet*. Using cluster statistical sampling, the authors estimated that 98,000 more Iraqis died than would have died in the absence of the war. Within this figure, ‘Violence accounted for most of the excess deaths and air strikes from coalition forces accounted for most violent deaths’ (Roberts et al., 2004).

Following extensive national media attention to this excess deaths study, on 17 November 2004, Foreign Secretary Jack Straw (2004) made a statement to Parliament. As part of it he said: ‘In many cases it would be impossible to make a reliably accurate assessment either of the civilian casualties resulting from any particular attacks or of the overall civilian casualties of a conflict. This is particularly true in the conditions that exist in Iraq.’ Thus not only did he contend that reliable figures did not exist; he also refuted the possibility of deriving them. Specific critiques made of the *Lancet* study related to the deficiencies of its data: their ‘limited precision’, the small sample size, the difficulties of accurately attributing responsibility for deaths and the problem of inferring civilian status.^6^

In choosing to question *The Lancet* study at the level of data in November 2004, it was possible for government ministers to discount its findings without questioning the statistical clustering method employed to calculate excess deaths—something technical advisors warned against in internal cross-departmental communications. Working at the level of data, however, at least opened up the prospect of reliable figures in the future—*if* the data could be improved. And yet, this also sat uneasily against the aforementioned contention also offered at the time that establishing reliable figures was ‘impossible’. Further complicating the sense of what was possible, in his Parliamentary statement, Foreign Secretary Straw (2004) also characterised deaths from hospital admissions as the most reliable information available.

Two years after the 2004 *Lancet* study came another prominent occasion for assessing the data on deaths. In 2006, *The Lancet* published a second survey by Johns Hopkins University. This one estimated that some 655,000 more Iraqis died than would have in the absence of the invasion up till 2006 (Burnham et al. 2006). In response, the Parliamentary Under-Secretary of State, Foreign and Commonwealth Office, Lord Triesman, said in the House of Lords:

My Lords, every civilian death is a tragedy and must be of concern in Iraq, as elsewhere. However, we continue to believe that there are no comprehensive or reliable figures for deaths since 2003. Estimates vary according to the method of collection. The figure of 655,000 given in the recent *Lancet* survey is significantly higher than other estimates, including those provided by the Iraqi Government. We believe that the Iraqi Government are best placed to monitor deaths among their own civilians (Triesman, 2006—see also Ingram, 2006).

This parliamentary statement drew attention to how it carried on with the official claims in 2004 that no reliable figures could be available. Yet, it differed in two respects. First, no reference was made to the underlying quality of the data of the 2006 survey (and, thereby, how the 2006 ‘survey’ redressed the central deficiencies previously pointed out by Foreign Secretary Straw in 2004). The reason for this *disregarding* was likely concerns raised in inter-ministry consultations. Documents obtained under the FoI Act indicated that those within the civil service repeatedly cited the improvements made to the quality of the underlying data in the 2006 study (Rappert, 2012b).

A second difference in the government response to the 2004 and 2006 studies related to the shift to what might be deemed as ‘methodological pluralism’ in the contention that ‘Estimates vary according to the method of collection’. This matter was later further spoken to in the House of Lords:

Lord Marsh: My Lords, does the Minister agree that the methodology of this study was unique in the way in which it was pursued? It is difficult to see how the Government can take the line, “The study was done in a way which is well known, and it was done very well, but we don't think that it is worth very much”.

Lord Triesman: My Lords, that is not the view that I have put at all. I said that there are different methods which have arrived at very different figures and that those methods also are legitimate. The way in which data are extrapolated from samples to a general outcome is a matter of deep concern and merits considerable study rather than the denunciation of one method compared with another.

As articulated in Triesman’s statements, the British government position was one of both *deferring* to the Iraqi government and *doubting* the possibility of sorting between what, in more recent parlance, might be called ‘alternative facts’.

The differences between figures on Iraqi dead derived from different methods would become a common topic of political and media commentary. The most prominent alternative attempt to derive figures was the “Iraq Body Count”. It relied on English language news stories as well as other substantiated reports to derive tallies through tabulating each confirmed reported civilian death. Over roughly the same time period as the 2004 *Lancet* study—the study that estimated 98,000 excess deaths—the Iraq Body Count tallied between 14,284 and 16,419 non-combatants deaths from military and paramilitary violence.

## Counting the dead

The previous section indicated some initial points of similarity and difference in the orientation to *death counts* associated with Covid-19 and the 2003 Iraq invasion. For both, excess deaths emerged within high level political debates as a prominent metric for gauging harm. For both, however, accountability in the here-and-now was side-stepped by British officials through their citing the lack of appropriate data to make assessments (and, subsequently in the case of Iraq, by also citing methodological variations). Promise was held out by state officials that something like a reliable understanding of deaths was at least potentially attainable at some future date that might allow for evaluating state action. In the case of Iraq, though, this potential was ambiguously situated against the stated impossibility of collecting ‘reliable’ data.

In this section, I want to unpack these headline similarities with regard to the status of *counts* by contrasting how the *counting* of deaths varied between the two cases. The possibilities for accountability through counting will be examined by considering the assumptions and choices associated with the inter-related matters of (1) the attention to what was being counted; (2) the resources mobilised in counting; and (3) the identified purposes of counting.

### What counting counts

While the aforementioned disparity between alternative Iraqi death counts was a frequent topic of note by British officials, much less commonplace was attention to differences in what was being counted. For instance, Lord Triesman’s October 2006 statement noted the contrast between the 2006 *Lancet* figure of 655,000 deaths and other tallies, yet without clarifying what those other tallies (such as Iraq Body Count) measured. What they measured was only a sub-set of what was captured by the *Lancet’s* all-encompassing excess death figure.^7^

The contrasts to the case of Covid-19 in relation to counting are stark. For instance, the circulation of multiple death counts pertaining to potentially overlapping categories was taken as a problem by state agencies from the start of the pandemic—one that needed to be remediated least public trust and the ability to track the pandemic suffer (see PHE, 2020c; ONS, 2020f). The result of this recognition was the progressive differentiation of categories of deaths, and modifications to the type of data analysis given in public reports.

Central categories of deaths were also revised in response to identified statistical limitations. Notably, for example, in the UK the primary sources for death counts were (1) the Department of Health and Social Care (DHSC) daily figures pertaining to those who tested positive for Covid-19, and (2) the Office of National Statistics (ONS) weekly figures derived from death certificates in which Covid-19 was mentioned. In weekly bulletins and one-off reports, the ONS broke down registered deaths by age, sex, region and place of death (e.g., ONS, 2020a, d) in order to assess the differential burden of the virus. What was covered by these sources changed in the spring of 2020 in response to publicly identified deficiencies. A high profile instance of this was the inclusion from April 29 of deaths outside of hospitals within the DHSC daily figures. For the ONS, revisions included efforts to measure deaths in care homes in England (ONS, 2020b), and to break down the causes of excess deaths beyond those formally linked to Covid-19 in death certificates in England and Wales, even as it was recognised this would be difficult to accomplish (ONS, 2020c).

**Fig. 2:**
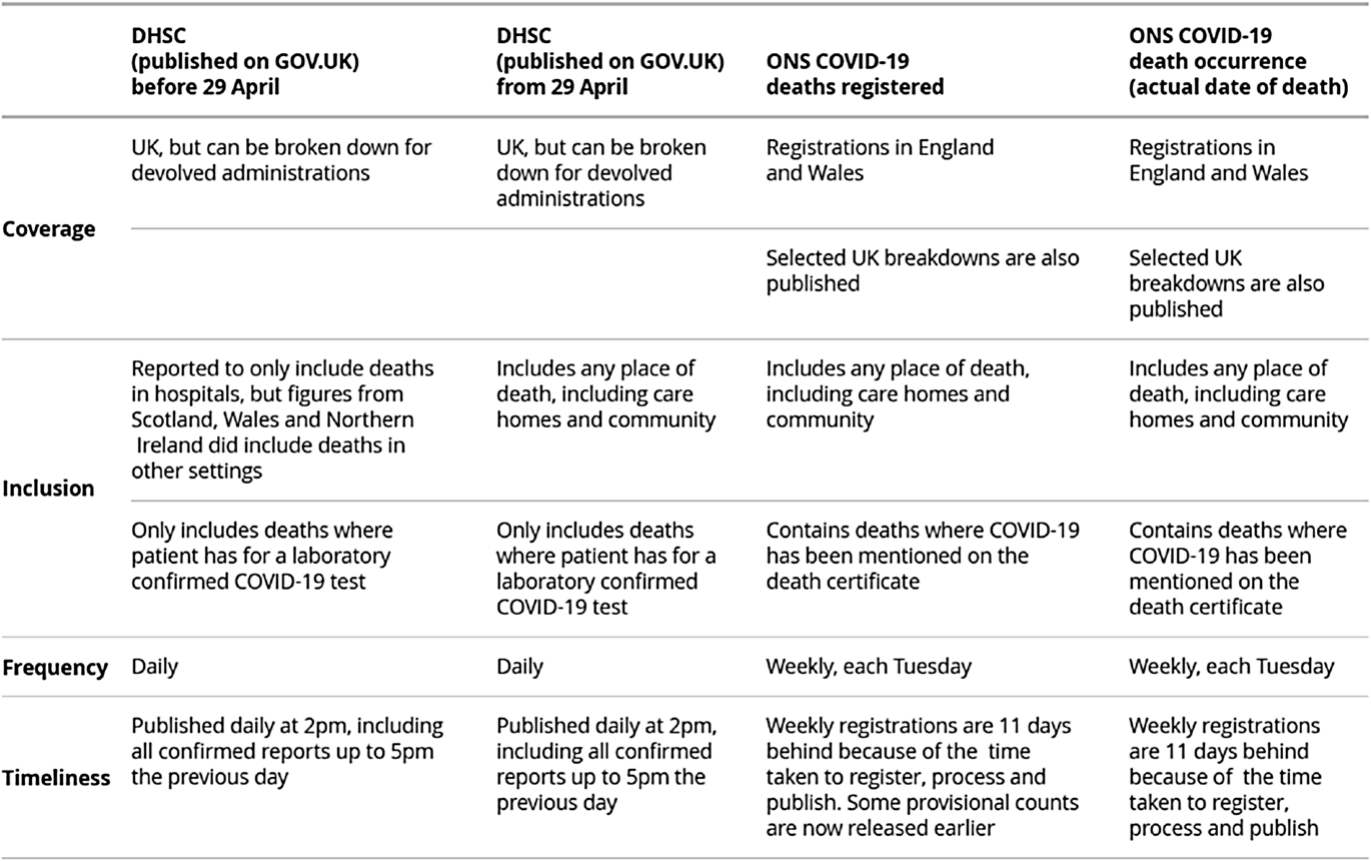
Office of National Statistics Breakdown of Types of Covid-19 Deaths (ONS, 2020f) (Reproduced under Open Government Licence.)

In relation to Covid-19, then, regard for what totals included and excluded led to reforms and refinements in what information was collected, what categories of deaths were relevant, and what information was made public. The combined result was a complex tapestry of sources offering varied perspectives in which unknowns about deaths were identified and redressed through reforms in the management and analysis of data (see Fig. 2).

By contrast, in the case of Iraqi deaths, the circulation of heterogeneous types of data was not taken by the UK as grounds for attempting to clarify, let alone to reform, British or Iraqi data management practices. Instead, repeatedly, the varied tallies served as ‘objections of *deconstruction*, figures impossible to verify and locate and therefore incapable of serving any intellectual operation other than that of the impossibility of determining their reality’ (Norris, 1994: 290). While officials cited an individual tally at certain times, this was done with hedging its ‘reliability’ (as in Straw, 2004). As such, data on deaths did not achieve the status of *immutable mobiles* (facts that could circulate while maintaining their integrity, Latour, 1987). More though, the lack of any internal or external efforts by the UK to engage with attempts to assess deaths meant that, for the British state at least, individual data sources did not take on the status of being *mutable mobiles* either*. * Unlike for Covid-19 deaths to British citizens, Iraqis could not benefit from statistical modifications to the codified traces of their deaths. In the absence of being treated as evidence for knowledge claims, it is questionable whether death tallies achieved the status of ‘data’ within the dealings of UK officials (Leonelli, 2016). Not being recognised as data proper meant there was little need to account for their implications.

### Mobilisation

In the case of Covid-19, reforms and refinements were enabled by the large-scale mobilisation of government departments and agencies that drew on pre-existing networks, resources and lines of authority as well as developed new networks, resources and lines of authority. For instance, in England, daily figures on confirmed positive tests would come to be derived from multiple sources: (1) hospitals use of a dedicated patient notification system, (2) Health Protections Teams from Public Health England through an electronic reporting system, and (3) comparing confirmed positive Covid-19 case lists held centrally in the pre-existent Second Generation Surveillance System (PHE, 2016) with health service patient records. Through combining multiple sources and subjecting them to semi-automatic cross-checks and quality assurances, the daily figures sought to provide rigorous information on all Covid-19 deaths.

In addition, transparency in the procedures for tallying death was sought in order to promote public confidence (PHE, 2020c) rather than only to be accurate. While geographically dispersed, the combined systems in place sought to function as ‘control zones’ (Lagoze, 2014) that could ensure the provenance and overall integrity of data through drawing on, and thereby reaffirming, established clinical systems.

But if re-enforcing existing ‘control’ mechanisms was a feature of mobilisation efforts, so too was the overhaul of previous epistemic conventions. For instance, the Office of National Statistics produced weekly figures derived from death certificates. Against mounting concerns that care home deaths related to Covid-19 were being missed, in April 2020 the ONS sought to gauge them in England (ONS, 2020b). To do so, it collaborated with the Care Quality Commission (CQC), an organisation that had collected data on deaths of care home residents. Prior to Covid-19, the CQC had not previously published data on deaths notified from care homes through its on-line reporting systems. One important feature of these notifications is that they ‘may or may not correspond to a medical diagnosis or test result, or be reflected in the death certification’ (ONS, 2020b). Thus, in making use of the CQC data for Covid-19 deaths, the ONS expanded the range of professional and occupational expertise as well as documentation that was treated as credible enough to inform its figures. Stated differently, through drawing on the notifications, the ONS integrated additional uncertainties into how deaths were counted.

In the case of Iraq, Lord Triesman’s call in 2006 for ‘considerable study’ about the methods for deriving casualty figures did not lead to the government commissioning such research or adopting externally derived figures (see Iraq Inquiry, 2016: 208–212). The official inquiry found little indication of efforts by the government either to assess direct deaths to civilians during the operations of British forces, or (as an occupying power) to improve the Iraqi systems responsible for recording deaths and issuing death certificates. A trial that had started in 2004 under the Cabinet Office to improve information on civilian deaths from military operations was halted before it was completed. Indeed, as it was argued by the Iraq Inquiry (2016: 218), much ‘more Ministerial and senior official time was devoted to the question of which department should have responsibility for the issue of civilian casualties than to efforts to determine the actual number’. Concerns about the quality of the data on deaths could have served to spur further official collection activities or attempts to bring together multiple sources. These concerns did not though. Instead, the portrayed difficulties with compiling figures was repeatedly taken as grounds against counting deaths at all.

In response to what it identified as limited state efforts, the official independent public inquiry into the invasion of Iraq called on the government ‘to make every reasonable effort to identify and understand the likely and actual effects of its military actions on civilians’ through working with NGOs and academics to establish the direct and indirect costs of war (Iraq Inquiry, 2016: 219). However, at the time of writing, no such efforts have been undertaken. Instead, echoing statements by officials made during the Iraq war against humanitarian concerns, the British state has made limited acknowledgement in its more recent military interventions that its forces could have caused even direct deaths to civilians (Airwars, 2018; Amnesty International, 2018). Efforts by the British government to gauge indirect fatalities and excess deaths associated with conflict remain further remote still.

### Purpose

One justification given for the lack of official studies of civilian deaths in the case of Iraq was their claimed lack of utility. For instance, the Ministry of Defence’s (MoD) Armed Forces Minister at the time of the invasion, Adam Ingram, argued in front of the Iraq Inquiry that establishing the number of Iraqi deaths would have not altered the military reality. This was so because the killing ‘was not being carried out by us on the civilians’ (Iraq Inquiry, 2016: 213). As Ingram further contended, if any deaths figures were to be calculated by the UK, this was not a job for his ministry.

If a sense of purpose was lacking in this respect for officials, it was lacking in other respects in the wider political and media debate. Just as which deaths were included in death counts was rarely specified, so too was the purpose of figures. In the case of armed conflict, these purposes can vary from memorialising suffering of innocents (a key aim of the Iraq Body Count), establishing assistance and reconstruction requirements, assessing the effectiveness of policies (a central objective in *The Lancet* studies), or revising use of force training and oversight. Yet, such purposes were seldom explicitly stated, let alone agreed between stakeholders, and certainly rarer still were the comparative merits of specific methodologies related to agreed purposes.

In contrast, Covid-19 death figures have not stood apart from the unfolding pandemic without any sense of how they might matter. They have served as milestones for marking and memorialising deaths, informed determinations of health care demands, and provided a means of assessing lockdown restrictions. As well, mortality figures deriving from institutionalised practices for determining the causes of death have been used to assess and revise those institutional practices. Such revisions have been intended to feed back into the production of mortality tallies. For instance, early on in the spread of the virus, concerns were raised that those in Black, Asian and Minority Ethnic (BAME) groups might face higher risks than those from White ethnic groups. In response to these concerns, and to wider ones about BAME health outcome disparities, the British government commissioned Public Health England to analyse surveillance data (PHE, 2020a). When this report was released, its lack of recommendations about what actions were needed led to widespread criticism (see, for instance, BMA, 2020). Criticism also followed the initial failure of the government to publish the findings on a consultation conducted with individuals and organisations within BAME communities (e.g., ITV, 2020). This resulted in extensive media speculation regarding the possible hidden motivations for the lack of publication in which this report’s fate was contextualised within wider ongoing national and international attention to the Black Lives Matter movement. When the consultation report was published ten days later, it not only called for further data collection and research into the biomedical, socio-economic, and structural determinants of health, but also for non-conventional forms of research (PHE, 2020b). In particular, the report stressed the need for community participatory research projects utilising local knowledge to ensure that research into the effects of Covid-19 informed concrete actions. Through such calls, the need to challenge existing ways of organising research entered into policy discussions in response to acts of counting.

It is important as well though to note what purposes counting did *not* serve. In the case of British lockdown policies in the first half of 2020, figures on deaths did not inform government formal cost-benefit calculations intended to justify the severity and duration of the restrictions placed on free movement. This situation differs from many other nation-state activities wherein the need to justify controversial policies has led to new methods to commodify and value bodies (Porter, 1995; Wernimont, 2018). Indeed, while standard health economic cost-benefit analyses would later come to inform British vaccination priorities, the policies for social restrictions have not been justified through cost-benefit analyses up until spring 2021.^8^ This has been the case despite some calls for such analysis within the governing political party for just such a cost-benefit elaboration (Spinney, 2021). Relatedly, efforts at counting deaths have not been marshalled as part of attempts to justify the ‘objectiveness’ of lockdown policies (cf. Porter, 1995).

## Leaks and cascades

Previous sections identified some of the similarities and differences between the ways that deaths were counted in relation to Covid-19 and the Iraq war as well as how death counts were evaluated. In the case of the Iraq war, British officials both refuted specific death counts as well as the ability to count deaths. In contrast, in the case of Covid-19, while considerable undertakings were made to count, accountability for tallied deaths has been rebuffed to date.

Following on from these sections, this one offers contrasting metaphors for characterising the relation between counts, counting and accountability: *cascades* and *leaks*. The intended relevances of these metaphors are two-fold. At one level, they are offered as conceptual shorthands for characterising data practices vis-à-vis state accountability for deaths. At another level, they are offered to set out a sense of the demands faced by scholars and others in conducting second-order analyses of state practices. In holding together these two levels, I want to advance further sensitivities and nuances for understanding the possibilities for accountability.

To begin with the first level, the metaphors of cascades and leaks speak to the relative overall differences in the quality of attention given to the dead by the British state. One way of breaking down this attention is through Laney’s (2001) data management typology relating to the *volume*, *velocity*, and *variety* of data. The *volume* of data produced in relation to the two cases differs dramatically. For Iraq, little effort was made by the state to tally data on who died directly or indirectly from the armed conflict and the data set produced by others were side-lined. In contrast, the management of data abundancy was a recurring demand for Covid-19.

Defining *velocity* as the speed at which data is accumulated and processed over time, in the case of Iraq the velocity of data was low. In the years that followed the invasion, the institutional apparatuses of the British state were almost exclusively limited to formulating sceptical evaluations of others’ intermittent efforts to gauge deaths (Rappert, 2012a; Iraq Inquiry, 2016). In contrast, between spring and summer of 2020, numerous reports, briefings, and datasets were produced in relation to Covid-19. Deficiencies identified in how official data were collected and reported brought revisions—such as determining contributory factors to specific categories of deaths (ONS, 2020e). The identification of deficiencies also brought the temporary suspension of the publication of daily DHSC figures between 17 July and 11 August. This suspension was initiated because of concerns that these tallies for England included individuals who tested positive for Covid-19, but subsequently died from unrelated causes. As a result of the review undertaken into the methods for tallying counts, the DHSC total for the England was reduced by 5337 deaths and new criteria were implemented to standardise counting across the nations of the UK. In this way, a tally derived at a particular point in time informed (or at least was expected to inform) subsequent activities; including what should circulate as data in the first place. Through such iterations, the counting of deaths could be characterised as the result of successive stages—stages that unfolded over a time span of weeks.

The ability to handle data *variety* (Laney, 2001), in the sense of mixing and managing heterogeneous types of data, provides another dimension for comparison. How to mix and manage data originating from multiple sources was an ongoing topic of explicit attention for Covid-19; one that led to various sequential refinements in data collection and analysis. In the case of Iraq, by contrast, officials positioned the availability of heterogeneous data and associated analyses as an insurmountable problem. Instead of serving as bases for the ever-refinement of inquiry, alternative data and analysis were orientated to as proof of the impossibility of deriving reliable knowledge.

To the volume, velocity, and variety of data management practice, the previous section suggests another dimension squarely related to political accountability: *value*.^9^ In particular, as a sub-set of value considerations, different orientations are evident across the cases in relation to the *urgency*. This term refers to the political imperative associated with attempts to produce knowledge. In the case of Covid-19, establishing the number of dead was treated as a responsibility of the state from the outset—a goal that was portrayed as necessary to pursue despite the complexities of doing so. Counting served as a basis for accountability in the taken-for-granted way the state had a duty to strive to protect its citizens. In addition, significant efforts were undertaken to ensure all deaths that could be counted were counted—be they deaths associated with Covid-19 directly or its secondary effects.

For those lives affected by the invasion of Iraq (an invasion partly justified as a means of liberating ordinary Iraqis), the contrast could hardly be starker. Counting was not treated as a duty of the British state from the start (or thereafter).^10^ Unsurprisingly then, little by way of resources were mobilised toward doing so.^11^ Moreover, the complications of deriving figures were regularly positioned as undercutting specific counts. The decidedly limited efforts made to compile information only pertained to deaths directly associated with British operations.^12^ As noted in the previous section, as seen by the state at least, the complied traces of deaths were arguably not even regarded as ‘data’ that needed to be accounted for.

Building on the identification of these highly contrasting configurations of the volume, velocity, variety, and value of data, I now want to sum up the differences between the cases by elaborating the notions of cascades and leaks.

The metaphor of *cascades* signals a situation wherein deriving and analysing data is regarded as crucial. More than just this though, cascade also signals the sequential modification of data and its analysis. In the case of Covid-19, a significant challenge faced by state agencies was how to manage the profusion of data and data types emerging over a relatively short period of time in order to present the most accurate and useful tallies possible. Counts of deaths at any one time could thereby inform subsequent actions. Overall, the identification of unknowns and uncertainties associated with how to count spurred further data generation efforts. However, the case of Covid-19 illustrates that establishing a dynamic pattern of counting need not led enable accountability. Indeed, at least to date, the very fluidity of counting efforts has been positioned by officials so as to undercut the need to account for death counts. As noted in Section Two, against the unfolding pandemic, claims were made that it was too early to evaluate deaths. As an inter-linked sequence of ongoing activities, the cascading together of data in relation to Covid-19 deaths served as grounds for forestalling calls to account for what had taken place.

In contrast, the metaphor of *leaks* signals an alternative set of practices and orientations. In the case of the invasion of Iraq, amassing data on deaths was not treated to as vital by officials; it was even regarded as a distraction. As such, no pattern of successive, iterative stages of data gathering and analysing was generated. As noted in Section Two, against emerging excess death counts, refutations were made against the possibility of counting deaths and the unreliability of any death counts. Instead of the identification of unknowns and uncertainties leading to the mobilisation of further resources and innovations as they had done in the case of Covid-19, for Iraq unknowns and uncertainties were treated by the British state over many years as standing against the possibility of establishing how many Iraqis died. In short, data on deaths led nowhere. The challenge of securing accountability from officials in these conditions stemmed from the limited amount of data available and the manner it was called into doubt—again and again.

### The conditions for second-order analysis

More than just speaking to the manner officials operated vis-à-vis accountability for deaths, I want to use the metaphors of cascades and leaks to speak to the varied conditions faced by analysts—scholars, journalists and others—seeking to offer second-order analyses of statecraft.

‘Leak’ provides an apt metaphor in the case of Iraq since, at least for many years following 2003, what was understood about internal government deliberations derived from small openings in access that were not planned. These included the unsanctioned release of documents to the media or official papers obtained through the FoI Act that come into force in 2005. Interpreting FoI documents brings a host of complications—a prominent one being partiality. While studies by the author prior to the Iraq Inquiry were able to draw on three sets of overlapping FoI requests during 2008–2010, in the end only some 48 emails, letters, and other documents (often moderately redacted) were obtained. Later, when the official Iraq Inquiry was given widespread access to official documentation and was able to call witnesses to testify, the limitations of the history that could be constructed previously became evident (cf. Iraq Inquiry, 2016; Rappert, 2012a).

The extent of the availability of information matters in the case of Iraq because of the way in which ministers and others forwarded refutations of derived tallies generated within a sequestered coterie. As a result, understanding the deliberative bases for these refutations was a central requirement in trying to establish how the UK acted in relation to concerns over Iraqi deaths. In this respect, whatever could be made visible about the internal deliberations—who knew what when and on what basis—added greatly to what was appreciated publicly.

Identifying disparities between what officials stated publicly on different occasions, and what was said in public statements and leaked deliberations, provided one means for analysts to try to surmise the inner workings of the state.^13^ With such disparities identified, at times explanations were forwarded by appealing to the kinds of ‘transparent political aims’ (Nettelfield, 2010: 167) often attributed to governments in armed conflict. For instance, the personal and political motivations of officials were marshalled to explain disparities between what was said in sequestered documents and in public forums (e.g., Horton, 2007).^14^ With so little ability to scrutinise the deliberations of British statecraft, the resort to motive- or interest-based explanations to bridge over gaps in what could be reconstructed was predictable.

Yet, the ability to discern the personal or shared group motives rests on the prospect of analysts being able to settle a variety of questions relating to how official documents should be interpreted. For instance, seeking to establish the real meaning the leaked or FoI documents implicated questions such as: How literal or readily decipherable are internal in-group communications? To what extent were officials deliberately crafting records with an eye to how they would later be made accountable for their (in)actions (Rappert, 2012a)?

In the case of Covid-19, with the emerging cascading of information, it is still possible to attribute underlying motives or interests to ministers and advisors (as well as the more general appeal to frontstage/backstage, inner/outer, public/private spheres). However, the extent of activity taking place across varied organisations within and outside of the state makes appeals to the motivations and wilful acts of individuals less salient, because the ability of officials to delimit or steer what is taking place is comparatively conditioned by others. Indeed, an important feature of the discussion of Covid-19 has been the manner it has involved public contests of a panoply of expertise that extends far beyond the central machinery of the state (Grundmann, 2020). In turn, that overt contest itself calls into question conceiving of a central task of secondary research as making visible the inner workings of the state. Instead, discernment is needed by analysts in determining what to examine and how to interpret it.

## Counts and accountability

The two previous sections highlighted points of *dissimilarities* between British responses to the Covid-19 pandemic and the invasion of Iraq in relation to practices of *counting*. In this final section, I want to return to *similarities* between the cases; both existent and possible.

Specifically, this will be done in relation to the widely anticipated (but as yet ill-defined) public inquiry into the handling of Covid-19 in the UK. As in the case of the Iraq war, public inquiries have long figured as prominent occasions for investigating the rights and wrongs of British statecraft. They have also been topics of contest regarding whether their scope, terms and powers have held governments to scrutiny (Public Administration Select Committee, 2005; Rolston & Scraton, 2005). In short, public inquiries are both truth telling and ignorance generating enterprises. As suggested previously regarding the Iraq Inquiry’s call for the UK to understand direct and indirect deaths, the likelihood that public inquiries result in meaningful reforms is a further matter for doubt.

By situating deaths with Covid-19 against the aforementioned dynamics associated with deaths in Iraq, I want to propose matters for attention regarding how accountability might figure in any such inquiry. An opening point for doing so is to underscore a contention underpinning the previous analysis: accountability is a situational and unfolding accomplishment, rather than a locked-in achievement. What kinds of justifications for actions are given, and whether justifications are treated as able to be given or necessary at all, emerge from the particulars of interactions. For instance, at the end of July 2020, the ONS (2020f) published a study comparing excess deaths across twenty-nine European countries between 3 January to 12 June. Within this report, England was identified as having the highest levels of excess mortality. Its publication, however, was not treated that day by the British Prime Minister in a news interview (Neilan, 2020) or Secretary of State for Health and Social Care in a speech (Hancock, 2020) as an occasion to draw (even provisional) international comparisons or evaluations of any kind related to death rates. Instead, both politicians spoke to fears prevalent during the end of July of a resurgence of Covid-19 elsewhere in Europe. Thus, while Covid-19 is a topic onto which substantial resources have been devoted, the quality and nature of the accountability realised depends on how evidence and argument are mobilised.

The previous analysis of the twists and turns with the dead of Iraq suggested how considerable state effort can be made to evade accounting for harms. The relevance of such experience for Covid-19 is a matter worthy of consideration. If it is taken to be the case that ‘in terms of mobilising the resources of the state, the pandemic has been as close as you can get to fighting a war without actually fighting a war’ (Hancock, 2020), then it is also conceivable that mobilisation of the state could extend to contesting its accountability for lives lost. This, too, is a common aspect of what it means to mobilise for war.^15^ In the previous sections, disregarding, doubting, deferring, deconstructing, delaying and displacing were some of the characterisations offered for how the possibility for accountability was foreclosed, diminished or sidestepped in relation to Iraqi deaths.

The imperative associated with *counting* in the case of the pandemic would suggest that many of the kinds of evasions prevalent in the case of Iraq could not apply to Covid-19. For the latter, the need for official agencies to count deaths has been a taken for granted duty. The situation might well prove more complicated, however, with regard to the standings of *counts*. This is so because, as illustrated through the cases examined here as elsewhere (Deringer, 2018), the imperative to engage in counting does not determine the import of counts.^16^

In closing, I want to forward future orientated concerns with accountability for Covid-19 deaths in a public inquiry—concerns that are sensitive to the scope for contrasting relations between data, knowledge and ignorance. I do so by proposing some of the ways in which accountability for deaths might be repulsed despite the considerable counting of deaths in the case of Covid-19:

*Ever-delay* As previous sections indicated, delay figured as a response to initial calls account for the number of Covid-19 deaths in the UK. The case of Iraq illustrates how attempts to hold the state responsible were rebuffed for years through arguing death counts were unreliable or otherwise in doubt. In the case of Covid-19, the wide-ranging nature of efforts to count could itself be marshalled to support a similar outcome. For instance, the imperative to definitively unpack the complexities of the biomedical, socio-economic, and structural determinants of death could be cited to forestall the possibility of political accountability till far beyond the time of any public inquiry.

*Prevalence* Previous sections outlined the significant differences in the amount and variety of data pertaining to deaths across the two cases. I suggested that these differences stemmed from starkly contrasting premises regarding the expected duties of the state. The prevalence of data and associated analyses in the case of Covid-19 might be taken as standing against the potential to contend that Covid-19 deaths cannot be derived. However, the case of Iraq illustrates how the availability of multiple and varied counts overtime were positioned by ministers to support the notion that it was not possible to derive reliable tallies. In the case of Covid-19, in the future, the ability to point to varied counts could function as situational stratagem for responding to criticisms or rebuffing calls for explanations.^17^ More subtly, such varied efforts could also be cited by officials to create a narrative that deaths cannot be established definitively. Thus, while every death would be acknowledged as a tragedy, the overall conclusion would be that comparative evaluations of the handling of the pandemic cannot be made with any real certainty.^18^

*Metrics* As expressed in the early stages of the epidemic, excess deaths were identified by officials as the metric for comparison across countries. However, the invasion of Iraq illustrates the ability of governments to radically shift metrics (for instance, recorded hospital deaths) in the face of inconvenient developments (Rappert, 2012b).

*Standards and Expertise* For deaths in Iraq, central characterisations of the data available—most notably its ‘reliability’—were marshalled in a manner that fostered ambiguity about what claims were being made and what evaluative standards were in operation. While considerable efforts have been undertaken to establish shared meaning of technical terms and categories related to the methods of determining Covid-19-related deaths, the standard for judging the validity of data remains open to contest. At the time of writing, for instance, the inability to establish the ‘true’, ‘exact’ or ‘absolutely firm’ death toll has served as a basis for some commentators to posit an irreducible ignorance regarding deaths (e.g., Lyons, 2020). Such a belief could be marshalled in any number of ways; for instance, to claim some without specialised knowledge (e.g., ‘the public’, journalists, and so on) cannot understand what statistics really mean (ibid.).

*Purpose* An appeal to true deaths tolls suggests an all-purpose number is possible in the first place. Against the multiple totals forwarded for Iraq, the inability to establish one definitive measure was positioned as scuppering the wisdom of trying to derive tallies at all. The suggestion that counts could vary depending on the purposes for which they served was rarely voiced. In the case of Covid-19, figures on deaths have had a complex relation to purpose. Numbers have been used to inform public health responses, but also to memorialise victims. To instil public trust, repeated efforts have been made to standardise what deaths within public reporting figures. Despite such efforts, uncertainty about the possibility of causally attributing deaths to Covid-19 has also been acknowledged. Such complexity provides ample ground for multiple and conflicting claims about what data matter, and the ultimate possibility for ‘adequately’ understanding deaths. Thus, a matter for the future is how to ensure the scope for plurality in purposes does not become a basis for substantially undermining the possibility for justifying actions.

## Conclusion

Efforts to determine the number of deaths associated with Covid-19 have been central to the response measures of many governments, NGOs and inter-governmental agencies. In part, assembling such data has been regarded as vital in holding governments to account for their actions and inactions. And yet, promoting political accountability through data is not at all straightforward.

By comparing two major cases of statecraft in which methodological concerns associated with deriving death tallies have featured prominently in public debates, this article has considered the fraught relation between data and accountability. It has done so through distinguishing the varied commitments and investments associated with counts and counting. What has been sought is a two way reflection. By contrasting the extensive efforts to count Covid-19 deaths against the scant efforts taken in the case of Iraqi dead, I have illustrated how efforts can be made to delimit democratic accountability. In addition, the strategies of officials to discount death counts in the Iraq war have served to warn how the British state might contest demands for accountability for Covid-19 related deaths. How such possibilities unfold in the years to come will be a measure of both democracy and data.

## Data Availability

Freedom of Information documents referred to in the text are available at https://brianrappert.net/publications.

## Footnotes

1 Added to this, the appropriateness of mortality figures to serve as a metric for loss or progress is fraught. Whether or not Joseph Stalin uttered the unsettling quote attributed to him—“A single death is a tragedy, a million deaths are a statistic”—the quantification of human suffering brings with it questions about the affective limits of abstracted representations (Wernimont, 2018).

2 The United Kingdom includes four individual nations: England, Scotland, Wales and Northern Ireland which are governed through a complex arrangement of UK-wide and devolved powers. For the purpose of this article, it is important to note that each nation has its own public health agency.

3 https://www.lexico.com/definition/accountable.

4 Records of these briefings are provided at: https://www.gov.uk/government/collections/slides-and-datasets-to-accompany-coronavirus-press-conferences.

5 The 48 emails, letters, and other documents obtained as part of three FoI requests are available at: https://brianrappert.net/publications.

6 Limitations identified by the Ministry of Defence’s Chief Scientific Adviser as part of inter-ministry consultations in response to publication of the report.

7 This lack of qualification was not just a feature of government statements. News reports and analysis in 2004–2006 and afterwards would highlight disparities between overall deaths counts to contend that establishing the number of dead was nearly impossible (e.g., Ellis, 2009; Steele & Goldenberg, 2008). Underpinning such assessments, however, was scant regard for what categories of deaths were being counted.

8 Although attempts have been made by economists to conduct cost–benefit analyses, see, for instance, Rowthorn and Maciejowski (2020).

9 For a discussion of value and knowledge, see Farquhar and Sunder Rajan (2014).

10 In large part because of the perceived absence of a *legal* requirement on the UK, see Iraq Inquiry (2016: 200).

11 This contrasts somewhat with the extent of attention to Iraqi casualties in the US, see Fischer (2010).

12 In the absence of significant involvement by major belligerent states, humanitarian NGOs have taken it upon themselves to develop internationally recognised standards for casualty recording. These efforts relate not only to the quality of data, transparency of how data is collected and analysed, but also call on groups to anticipate how their recording efforts might cause harm (Every Casualty, 2016).

13 As an instance of how government portrayals varied over time, contrast Symons (2004a, 2004b) regarding the reliability of hospital figures.

14 Such efforts were aligned with a “methodological individualism” that takes individuals to be intentional, rational, and calculating (as in Thomas, 2017).

15 For one case of how counts of deaths were mobilised, see Dyson and Cetorelli (2017).

16 Likewise, the relationship between data on deaths and accountability for actions is indeterminate. For instance, Western governments have taken it as a core obligation of the state to determine the number of their own fallen military personnel in war. Rather than mounting deaths being a reason for re-assessing state action, though, the reporting of deaths has been marshalled by officials in an operative manner to impress on citizens the aims of statecraft and the wartime agendas of political leaders (e.g., Wagner, 2019).

17 Arguably it has done so already, see Hansard (2020a: Column 548).

18 See Proctor (2011) for a historical examination of utility of these kinds of arguments.
